# Effects of Folinic Acid Administration on Lower Limb Ischemia/Reperfusion Injury in Rats

**DOI:** 10.3390/antiox10121887

**Published:** 2021-11-25

**Authors:** Iñigo Cearra, Borja Herrero de la Parte, Inmaculada Ruiz Montesinos, Ana Alonso-Varona, Diana Isabel Moreno-Franco, Ignacio García-Alonso

**Affiliations:** 1 Osakidetza Basque Health Service, Department of Traumatology and Orthopedics, Basurto University Hospital, ES48013 Bilbao, Spain; 2 Department of Surgery and Radiology and Physical Medicine, Faculty of Medicine and Nursing, University of the Basque Country UPV/EHU, ES48940 Leioa, Spain; mariainmaculada.ruiz@ehu.eus (I.R.M.); ignacio.galonso@ehu.eus (I.G.-A.); 3 Regenerative Therapies, Osteoarticular and Tendon Pathology Research Group, Biocruces Bizkaia Health Research Institute, ES48903 Barakaldo, Spain; 4 Interventional Radiology Research Group, Biocruces Bizkaia Health Research Institute, ES48903 Barakaldo, Spain; 5 Department of Gastrointestinal Surgery, Donostia University Hospital, Osakidetza Basque Health Service, ES20014 Donostia, Spain; 6 Department of Cell Biology and Histology, Faculty of Medicine and Nursing, University of the Basque Country UPV/EHU, ES48940 Leioa, Spain; ana.alonsovarona@ehu.eus; 7 Osakidetza Basque Health Service, Department of Vascular Surgery and Angiology, Basurto University Hospital, ES48013 Bilbao, Spain; dianaisabel.morenofranco@osakidetza.eus

**Keywords:** ischemia-reperfusion injury, lower limb, tourniquet, folinic acid, prophylactic treatment, functional recovery

## Abstract

Surgery under ischemic conditions, lasting up to 3 h, is routinely performed in orthopedic surgery, causing undesirable injury due to ischemia-reperfusion syndrome, with short and medium-term functional repercussions. To date, there is no established prophylactic treatment. In this work we evaluated folinic acid (FA) in a rodent model of lower limb ischemia-reperfusion (IRI-LL). 36 male WAG rats underwent 3 h of lower limb ischemia. In the saline group, rats received intraperitoneal administration of saline (used as vehicle for treatment). In the experimental group, rats were pretreated with FA (2.5 mg/kg) before the end of ischemia. After ischemia, animals were sacrificed at 3 h, 24 h or 14 days (for biochemical determination (Na+, K+, Cl-, urea, creatinine, CK, LDH, ALP, ALT, and AST), pathological assessment, or functional study using the rotarod test; respectively). Another six animals were used to establish the reference values. The prophylactic administration of FA significantly reduced the elevation of biochemical markers, especially those that most directly indicate muscle damage (CK and LDH). In addition, it also improved direct tissue damage, both in terms of edema, weight, PMN infiltrate and percentage of damaged fibers. Finally, the administration of FA allowed the animals to equal baseline values in the rotarod test; what did not occur in the saline group, where pre-ischemia levels were not recovered. Following 3 h of lower limb ischemia, FA minimizes the increase of CK and LDH, as well as local edema and leukocyte infiltration, allowing a faster recovery of limb functionality. Therefore, it could be considered as a prophylactic treatment when tourniquet is used in clinics.

## 1. Introduction

A common practice in extremity surgery, especially in orthopedic surgery, involves the application of controlled ischemia, commonly performed through the application of a pneumatic cuff as a tourniquet. This procedure provides a blood-free surgical field, which facilitates both anatomical dissection and identification of structures. However, these cuffs, while making surgery easier, also cause injury to the limb to which they are applied. This injury arises from the combination of three different factors: a) mechanical damage, due to the pressure of the pneumatic cuff on the tissues; b) anoxic damage, due to oxygen deprivation resulting from ischemia; c) inflammatory-oxidative damage, due to reperfusion when the pneumatic cuff is released. The latter two are intimately linked, and both may be grouped under the so-called ischemia-reperfusion injury (IRI), a phenomenon resulting from the reoxygenation of tissues previously subjected to anoxia. Blood flow restoration triggers a sequence of biochemical processes, such as the formation of reactive oxygen species (ROS), activation of cytokines, and alteration of capillary permeability, which exacerbate the cellular dysfunction suffered during the ischemic process, being the muscle the most affected tissue in the extremities [1,2]. In this context, the use of the ischemia cuff has been associated with a worse clinical recovery at least in the short and medium term (increased pain, edema, quadriceps weakness...) [3,4,5,6,7,8], which has even led some authors to question the risk-benefit balance of using the ischemia cuff in knee arthroplasty [9].

In order to minimize the cuff pressure-induced damage, not only improvements have been introduced in terms of cuff shape and width [10], but also in the pressure setting to the minimal arterial occlusion pressure (MAOP) [11]. However, this only has an effect on limiting or eliminating the mechanical damage previously described, while not addressing the ischemic, inflammatory, and oxidative damages, which trigger IRI.

Both clinically and experimentally, different molecules have been proposed for the prophylactic pharmacological treatment of IRI: (a) antioxidant compounds, such as equivalents of superoxide dismutase (SOD), malondialdehyde (MDA), allopurinol, melatonin, N-acetylcysteine (NAC) [12,13,14,15]; (b) statins [16,17]; and (c) anesthetics, such as propofol, sevoflurane, dexmedetomidine or oxycodone [18,19]. Another proposed therapeutic approach is the so-called “ischemic conditioning”, first described in myocardium [20]. This maneuver consists of performing one or several short-term ischemia periods, either in the same or different anatomical location, and before or after the long ischemia period. The basis of this approach lies in the activation of endogenous redox buffer mechanisms, thus avoiding the need of exogenous antioxidants. There is no defined protocol described for the performance of conditioning, at least in the extremities [21,22]. In most of the studies, the effectiveness of the abovementioned treatments is assessed by considering only immediate indicators of oxidative stress, such as MDA; there are very few experiments including biochemical, anatomopathological or clinical markers of muscle damage with a later onset [13,14,16,17,23]. We have recently validated a rat model in which, by exerting a controlled pressure to achieve 3 h of ischemia in the lower limbs, we are able to assess the response to IRI from the biochemical, histological and functional point of view [24].

Folic acid, or its active form, folinic acid (FA), is a well-known antioxidant [25,26,27], inexpensive, and without described toxicity [28]. In spite of this, only a few works are available using it to treat IRI, either at the level of the cardiac muscle [29], or in the mesenteric and hepatic tissues, where the administration of 2.5 mg/kg of FA showed a protective effect [30,31].

This piece of work aims to evaluate the effectiveness of FA as a treatment for lower limb IRI (IRI-LL), in a rat model emulating the characteristics, techniques and duration of ischemia periods used in clinical practice when performing limb surgery under ischemic conditions, by analyzing biochemical, anatomopathological and functional parameters considered to be analogous to those used in clinical practice.

## 2. Materials and Methods

All procedures were performed in accordance with current European and Spanish legislation and were approved by the Ethics Committee on Animal Experimentation of the University of the Basque Country (ref.: M20/2015/075/ Herrero de la Parte). 

For the present work we used male WAG/RijHsd rats, aged 3–4 months and with an average weight of 302 ± 12 g, bred and maintained in the Animal Facility of the University of the Basque Country, UPV/EHU, with food and water “ad libitum”, and a 12 h light-dark cycle and constant temperature of 24 °C. In order to minimize the variability due to the circadian rhythms of the animals, all the procedures performed for the induction of IRI-LL were carried out in the morning.

A total of 42 animals were randomly distributed in two experimental groups (18 animals each), with three different sacrifice times (3 and 24 h, and 14 days), and a control group (six animals). The ischemia was always performed on the right lower limb.

### 2.1. Induction and Maintenance of Lower Limb Ischemia

Ischemia was induced and maintained by an in-house designed and built mechanical device. Briefly, it consists in an adjustable support that allows placing the anesthetized animal in the supine decubitus position and a nylon cable, connected to a dynamometer and a plunger system, which slides around the limb at its base exerting a tension of 1 kg, the whole assembly being stabilized by two articulated arms [24].

The animals were intraperitoneally anesthetized with diazepam (15 mg/kg), ketamine (80 mg/kg) and medetomidine (0.5 mg/kg) and placed in the device in the supine position. The cable connected to the dynamometer was placed around the right lower extremity and kept elevated with the help of an auxiliary arm. Once the leg was exsanguinated by an elastic bandage (Esmarch bandage), the tension of the dynamometer was set at 1 kg to maintain the limb under ischemia. The correct achievement of ischemia was verified by checking the absence of capillary filling, the paleness of the limb and verified by a laser Doppler probe over the pedial artery territory. After half of the ischemia time (80–100 min), a new dose of diazepam (7.5 mg/kg) was administered to maintain the animal’s sedation until the end of the 3-h ischemia period. The animals of the treated groups, were given FA (2.5 mg/kg, ip) 20 min before the end of the ischemia period. The same volume of saline was administered to animals in the control groups.

Once the 3 h of ischemia were completed, the tension of the tourniquet was completely released, allowing reperfusion of the limb during the period corresponding to each experimental group. Restoration of blood flow was verified by the presence of clinical signs of reperfusion and laser Doppler scanning. Post-ischemia recovery was accomplished under a heat source at 22–24 °C, and buprenorphine (0.05 mg/kg sc) was administered to minimize pain. The animal’s condition was checked every 30 min, until complete recovery.

### 2.2. Biochemical Determinations

Serum parameters changes were analyzed after 3 h of reperfusion. For this purpose, the animal was anesthetized with 1.5% isoflurane and the entire volume of circulating blood (5–7 mL) was obtained by puncture of the abdominal cava vein with a 20 G needle. The blood obtained was centrifuged at 3000 rpm for 10 min in SST ™ II Advance tubes (BD Vacutainer^®^, East Rutherford, NJ, USA). The serum obtained was aliquoted and stored at −25 °C until analysis. 

The determination of all biochemical parameters was performed on a Cobas^®^ 8000 module c702 (Roche Diagnostics GMBH, Mannheim, Germany). Electrolyte levels [sodium (Na^+^), potassium (K^+^) and chlorine (Cl^−^)], renal function indicators (urea and creatinine), ubiquitous enzymes [alkaline phosphatase (ALP), aspartate transaminase (AST) and alanine transaminase (ALT)] and muscle enzymes creatine kinase (CK) and lactate dehydrogenase (LDH) were quantified.

### 2.3. Histopathological Study

After allowing 24 h of reperfusion, tissue damage was evaluated. Macroscopically, edema was assessed by measuring the perimeter of the limbs and weight of the gastrocnemius muscle of both lower limbs (left limb as a control and right limb subjected to ischemia). The perimeter was measured at the proximal level of the thigh with a tape measure. This measurement was performed after shaving the animal’s limb, with the animal placed on the ischemia device, and the limb with a slight traction to the zenith through superior auxiliary arm. Afterwards, both control and ischemic gastrocnemius muscle were removed, weighed and fixed in 4% formaldehyde.

For microscopical evaluation of the damage, five randomly chosen slides were counted for each set of gastrocnemius muscles examined from each animal [32]. The histological section were stained following standard hematoxylin-eosin staining protocol and then observed under an optical microscope (at 20× magnification). Polymorphonuclear neutrophil (PMN) infiltration was also assessed, by counting the number of these cells in each field.

### 2.4. Functional Analysis

Six rats per group were tested on a Rotarod apparatus (Panlab Harvard Apparatus, Barcelona, Spain). The rats were placed on a moving rod with a rotation speed of 15 r.p.m. Following the 15 s acclimation time, the Rotarod was gradually accelerated on a 30-s slope, which increased the speed from 15 r.p.m. to 25 r.p.m.

Once the rod acceleration started, the latency to fall time started to be recorded automatically by the SeDaCom v2.0.03 software (Panlab Harvard Apparatus, Barcelona, Spain); the speed of the rotarod at the time of fall was also recorded. Prior to ischemia induction, animals were subjected to a 2-day training protocol before being tested on the day −1, which acclimated the rats to the rotarod. The animals were subjected to the Rotarod test every day during the first week after ischemia, and also on day 10 and day 14.

### 2.5. Statistical Analysis

Statistical processing of the results was carried out with Prism^®^ 7 statistical software (GraphPad Software, San Diego CA, USA). First, the normal distribution of the data to be studied was verified using the Kolmogorov-Smirnov normality test. Once it was verified that the data complied with a normal distribution, the results were expressed as the mean and standard deviation (SD) and parametric tests were performed between the different experimental groups.

Analysis between the limbs subjected to I/R and their healthy contralateral was performed using a Student’s t-test for paired samples. The analysis between the treated and untreated groups was also performed using a Student’s t-test, in this case for unpaired samples. For all analyses, statistically significant differences were accepted when the *p*-value was < 0.05.

## 3. Results

### 3.1. Ischemia Induction

The procedure to induce ischemia was successfully performed and well tolerated in all the rats, without any observable adverse effect. No events of pressure loss were reported related to the device used for induction. The pressure applied (1 kg) remained constant during the 3 h of ischemia. In those animals that were sacrificed after 24 h and 14 days, no adverse effects related to the tourniquet were evident after ischemia. No signs or symptoms of pain or suffering were reported.

### 3.2. Effect of FA on Biochemical Parameters Analyzed in Serum Samples

**Figure 1:**
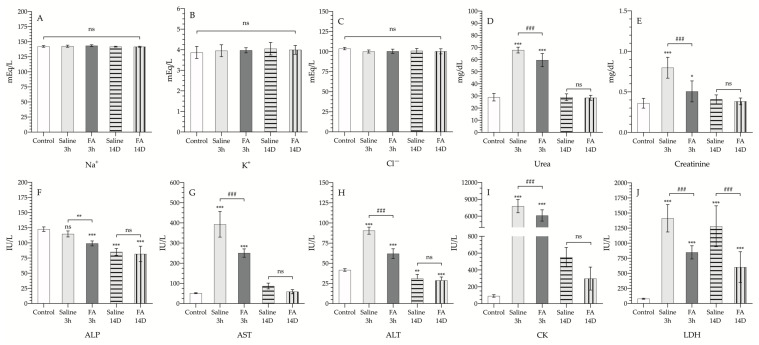
Serum electrolytes and enzymes of control animals (control, white bar); animals exposed to 3 h of ischemia & 3 h of reperfusion, and treated with saline (saline 3 h, gray bar), or treated with folinic acid (FA 3 h, dark gray bar); animals exposed to 3 h of ischemia & 14 days of reperfusion, and treated with saline (saline 14 D, gray bar with horizontal striped pattern), or treated with folinic acid (FA 14 D, gray bar with vertical striped pattern. Na^+^ (**A**), K^+^ (**B**), Cl^−^ (**C**), urea (**D**), creatinine (**E**), alkaline phosphatase (ALP (**F**)), aspartate transaminase (AST (**G**)), alanine transaminase (ALT (**H**)), creatine kinase (CK (**I**)), and lactate dehydrogenase (LDH (**J**)). Units are expressed in international units per liter (IU/L), milligrams per deciliter (mg/dL) or in milliequivalents per liter (mEq/L). The asterisks show the statistically significant differences compared to the control (* *p* < 0.05; ** *p* < 0.01; *** *p* < 0.001), and the pads show the statistically significant differences between the groups indicated by the upper box (### *p* < 0.001); ns: *p* > 0.05.3.3. Effect of FA on histological damage induced by IRI.

The results of the biochemical parameters studied are shown in Table 1 and Figure 1. Both in the animals treated with FA and in the vehicle group, the damage after ischemia and reperfusion did not lead to significant alterations in the levels of Na^+^, K^+^ or Cl^−^, neither after 3 h of reperfusion nor after 14 days.

**Figure 2:**
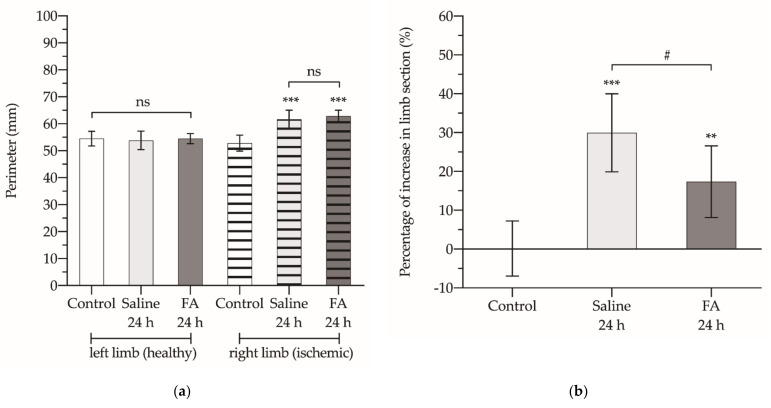
Perimeter (**a**) and percentage of increase in the cross-sectional area of the ischemic limb with respect to the contralateral healthy limb (**b**). The asterisks show the statistically significant differences compared to the control (** *p* < 0.01; *** *p* < 0.001), and the pads show the statistically significant differences between the groups indicated by the upper box (# *p* < 0.05); ns: *p* > 0.05.

**Figure 3:**
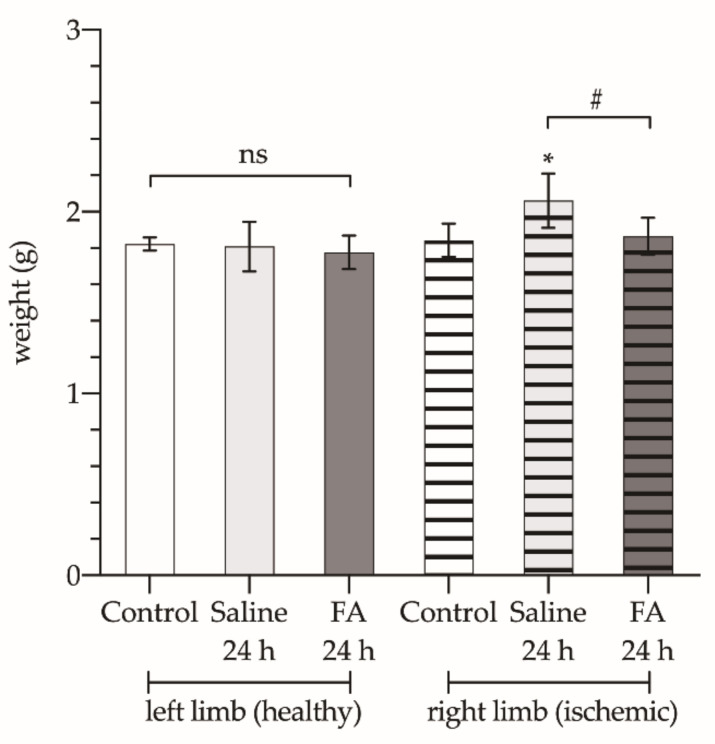
Fresh weight of the gastrocnemius muscle of both limbs: left limb (healthy, bars without pattern) and right limb (ischemic, bars with vertical striped pattern), from animals not subjected to ischemia (white bars), treated with saline (Saline 24 h, light gray bars), or treated with folinic acid (FA 24 h, dark gray bars). The asterisks show the statistically significant differences compared to the control (* *p* < 0.05), and the pads show the statistically significant differences between the groups indicated by the upper box (# *p* < 0.05); ns: *p* > 0.05.

**Figure 4:**
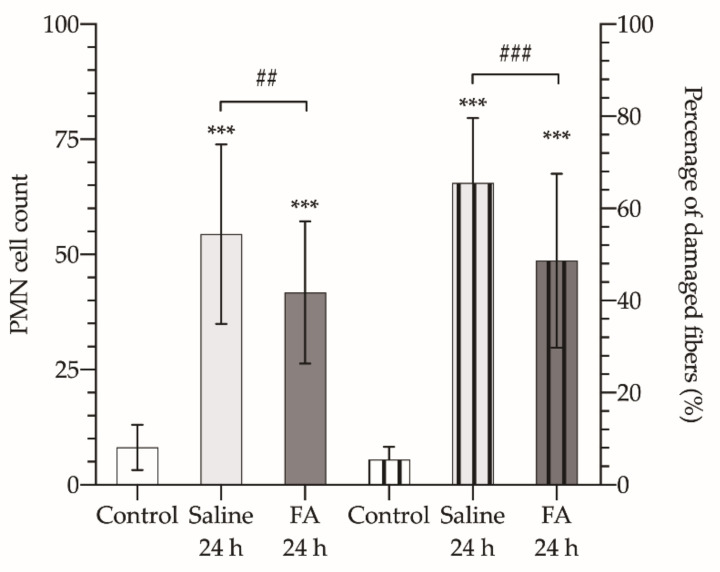
Polymorphonuclear neutrophil (PMN) cell count (left axis, bars without pattern) and percentage of damaged fibers in each field at 20 × (right axis, bars with vertical striped pattern) of control healthy animals (Control, white bars), and animals undergoing 3 h of ischemia and 24 h of reperfusion and treated with saline (Saline 24 h, light gray bars), or treated with folinic acid (FA 24 h, dark gray bars). The asterisks show the statistically significant differences compared to the control (*** *p* < 0.001), and the pads show the statistically significant differences between the groups indicated by the upper box (## *p* < 0.01; ### *p* < 0.001); ns: *p* > 0.05.

The histopathologic results are detailed in Table 2 and presented in Figure 2, Figure 3 and Figure 4.

Regarding urea and creatinine levels (Figure 1d,e), a significant increase was observed after 3 h of reperfusion, up to 66.3 ± 5.06 mg/dL and 0.798 ± 0.13 mg/dL, respectively. This increase was significantly lower in those animals receiving FA treatment prior to the start of reperfusion (59.6 ± 6.13 mg/dL and 0.506 ± 0.13 mg/dL, respectively) (*p* < 0.001). Both analytes’ levels returned to normal levels 14 days after controlled ischemia (*p* > 0.05).

On the other hand, it was also observed that pretreatment with FA of animals exposed to 3 h of reperfusion resulted in a significant decrease in ALP values (99 ± 4.15; *p* < 0.001) (Figure 1f), compared to the control group. This decrease in ALP levels was sustained over time until the 14th day, both in the animals that received saline and in those treated with FA (85.2 ± 5.46 and 81.8 ± 12.8, respectively); without evidence of any differences between untreated and treated animals (*p* > 0.05). Both transaminase levels (AST and ALT, Figure 1G-H) experienced a marked increase after 3 h of reperfusion, the increase being significantly lower in those animals that received prophylactic FA treatment; after 14 days, AST and ALT levels returned to values similar to those detected in healthy animals that had not undergone the IRI process.

Finally, CK and LDH levels (Figure 1i–j), enzymes that are mainly indicative of muscle damage, were significantly increased after three hours of reperfusion in the saline group. However, the animals that received FA showed a significantly lower increase in serum levels of both enzymes (6123 ± 1000 vs. 7792 ± 1187 IU/L, and 848 ± 109 vs. 1413 ± 227 IU/L, respectively). When analyzed on day 14th, CK values were significantly decreased, through still more than doubled the control values (*p* > 0.05). On day 14th, LDH values in the saline group remained significantly elevated compared to the animals not undergoing ischemia (control group), whereas treatment with FA reduced serum LDH values by half.

After 3 h of ischemia and 24 h of reperfusion, no changes were found in the circumference of the contralateral healthy limb (not subjected to ischemia). However, a significant increase in the perimeter of the limb undergoing ischemia was observed, without finding significant differences between the animals that received saline or FA (Figure 2a). But when determining the percentage of increase in the cross-sectional area of the ischemic limb with respect to the contralateral healthy limb, we found that this value in the animals treated with saline was 30%, while in those treated with FA it was only 17% (*p* < 0.05) (Figure 2b).

Regarding the weight of the gastrocnemius muscle (Figure 3), there are no differences between the animals of the three groups (control, ischemic without treatment, and ischemic + FA). In the limbs undergoing ischemia the mean weight of the gastrocnemius muscle was significantly increased (2.06 ± 0.150 g vs. 1.84 ± 0.092 g) (*p* < 0.05). However, when FA was administered before restoring blood perfusion, this increase in the muscle weight was prevented (1.86 ± 0.102 g; *p* > 0.05).

On the other hand, after microscopic observation of histological sections of the muscle, it was found that, after 3 h of ischemia and 24 h of reperfusion, there was an increase in the PMN cell count compared to control muscles (8.133 ± 4.93 PMN/field, Figure 4). In those animals that were not treated with FA, the PMN count increased by 6-fold, up to 54.44 ± 19.47 PMN/field, while FA treatment made this increase barely reach 5-fold (41.75 ± 15.43 PMN/field), resulting in a statistically significant reduction (*p* < 0.01). Which translated into a cell density of 4.52 × 10^4^ PMN/mm^2^, and 3.42 × 10^4^ PMN/mm^2^, respectively.

Finally, by counting the damaged fibers and calculating their percentage with respect to the total number of fibers per field at 20×, we found that FA also showed a protective effect against muscle cell damage. The percentage of damaged fibers in those animals treated with FA did not reach 50% of the fibers observed in the field, while in those that received saline, the damaged fibers raised over 65% (*p* < 0.001).

### 3.3. Effect of FA on Functional Recovery

**Figure 5:**
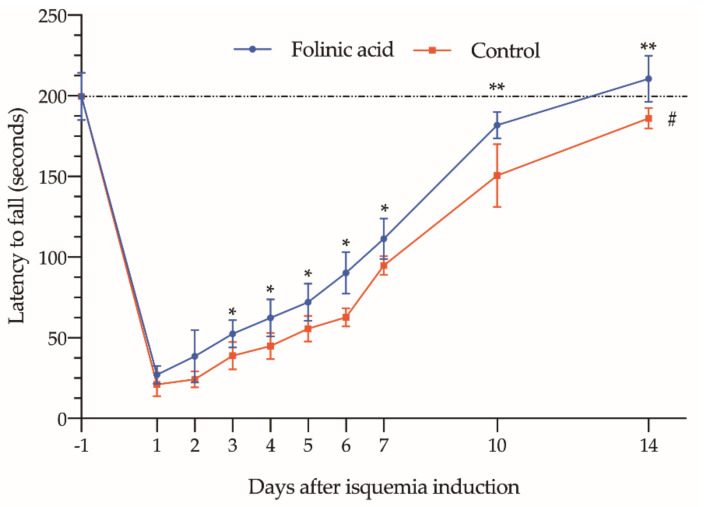
Assessment of limb functional status over a 14-day period using the Rotarod test. Data show the mean and standard deviation in the fall time (expressed in seconds) of animals subjected to 3 h of ischemia period, treated with folinic acid (blue line) or with saline (red line). The asterisks show the statistically significant differences compared to the control (* *p* < 0.05; ** *p* < 0.01). The dashed red line box indicates the differences between the 14th day of the study and the baseline values determined on the day before ischemia induction (day −1) (# *p* < 0.05; ns: *p* > 0.05).

Finally, when taking into account data recorded in the Rotarod test, we can observe that the pre-treatment with FA significantly increased “latency time to fall” (Figure 5), expressing an improvement in lower limbs functional capacity. This effect could be seen as early as the third day after induction of ischemia, showing 20–40% higher figures in those receiving the treatment (*p* < 0.05 compared to ischia without treatment). 

On the 14th day, only those animals treated with FA recovered a normal “latency time to fall”, similar to those obtained before ischemia (210.5 ± 14.42 vs. 199.6 ± 14.76 s; *p* > 0.05). In animals that did not receive any treatment, the “latency time to fall” was still significantly shorter (186 ± 6.39 vs. 199.6 ± 14.76 s; *p* < 0.05).

## 4. Discussion

Cuff-induced ischemia is a procedure commonly used in many surgical procedures, particularly in orthopedics. This technique implies an inherent local damage to the limb to which it is applied, with an impact on clinical parameters [4,5,6,7,8]. Nowadays some efforts have been made to control this local damage, including improvements in cuff design and recommendations for its use [10,11]; however, no treatment or prophylaxis for IRI-LL are currently provided.

In the present study, we have analyzed a prophylactic treatment with FA in a model of IRI-LL, induced by 3 h of ischemia. This time, although longer than that usually applied in clinical reality [33,34], has been chosen to ensure that, even in those long-term surgeries, FA could be indicated as a preventive treatment for IRI-LL. Furthermore, experimental and clinical evidence at this period of ischemia indicates that the damage remains located in the limb [24,35,36,37].

The device used to induce ischemia differs from that published in rat models. However, we consider that our system, by having a device to measure the pressure exerted by the tourniquet, is closer to those used in clinical practice [21,22]. These invasive systems, which require more aggressive procedures on the animal [21,22], are not suitable as a proper model of IRI-LL. Similarly, non-invasive systems lacking devices to control the exerted pressure (rubber bands, tubular rings), more closely related to the Esmarch band model [17,32,38,39,40,41,42], are also far distant from clinical practice, as they are more similar to a tourniquet than to the current pneumatic cuff inflated at MAOP.

To assess the potential therapeutic effect of FA, we have chosen biochemical parameters that are widely used in clinical practice (AST, ALT, CK, LDH, ALP, creatinine and ions such as K^+^, Na^+^ and Cl^−^), and also commonly used in several models of IRI, both in experimental and clinical studies [38,43,44,45,46,47,48]. Specific oxidative stress markers provide important information at the experimental level. But at the clinical level many of them still need to be validated in larger sample sizes and compared to current clinical standards in order to be considered as valid parameters in clinical diagnosis [49]. This is the reason for ruling out other specific oxidative stress markers such as ischemia-modified albumin (IMA), isoprostanes-iosfurans, or MDA [13,15,18,50,51]. In addition, we also perform pathological analyses to assess the degree of muscle fiber destruction and PMN infiltration. Finally, the rotarod test was used to evaluate mid-term recovery by measuring the functional status of the limb subjected to ischemia.

Reviewing the literature, no studies have been found that analyze the effects of FA as a treatment for IRI-LL. However, published studies by other authors, and by ourselves, have shown that FA can be used to prevent IRI in other tissues such as the ovary, kidney, liver, intestine, etc. [52,53,54,55,56]. In all these studies, FA improved, as in the present study, the anatomopathological damage to the tissue as a consequence of IRI.

Focusing on IRI-LL, other authors have studied compounds as the C5a receptor antagonists, which blocks the effects of C5a, a potent anaphylatoxin with key roles in the inflammatory and immune response [44], or N-metyl-4-isoleucine-cyclosporin (NIM-811), a mitochondria specific drug, which can prevent IRI, by inhibiting mitochondrial permeability transition pores (mPTP) [45].

Woodruff et al. [44] demonstrated that C5a receptor antagonists were able to decrease the levels of those enzymes that were markers of muscle damage. CK and LDH values were 3 and 4 times lower after pre-ischemia or pre-reperfusion administration of C5a receptor antagonist. AST, ALT, and creatinine also showed a similar response when animals were pre-treated (3-, 1.5- and 1.4-times lower levels, respectively). Garbaisz et al. also observed a favorable response when animals were pre-treated with NIM-811. Their results showed a decrease in damage more in line with that described in this paper. In addition, histological assessment of muscle fibers showed a 4-fold increase in fiber viability in treated animals (7.94% ± 5.71% vs. 36.97% ± 11.06%) [45]. Indeed, hyperkalemia was diminished.

Non-pharmacological therapies, such as remote ischemic postconditioning, investigated by Gao et al. were also shown to reduce rhabdomyolysis damage (determined by CK and LDH levels) in a model of myocardial IRI [46]. This reduction was demonstrated both in anatomopathological parameters, such as the reduction of the infarct area or the percentage of apoptosis (22.9 ± 3.3% vs. 40.9 ± 6.2% and 13.4% ± 3.1% vs. 26.2% ± 3.1%, respectively), and in serum CK and LDH levels (21.97 ± 4.08 vs. 35.86 ± 2.91 ng/mL and 6.17 ± 0.58 vs. 8.37 ± 0.89 U/mL).

The therapies analyzed in these studies were able to improve the markers of IRI-LL damage in different ways. Although it is true that data reported by these studies are not quite homogeneous, it is due to the different IRI-LL models used. For example, as different ischemia and reperfusion periods were used, it led to different degrees of initial damage. Furthermore, the induction devices also differed greatly between those studies and this piece of work. In addition, the routes used to administer the compounds used to treat IRI-LL are also very heterogeneous. It is therefore difficult to assess which of them is more effective, as there are many factors that could influence the analysis.

It does appear that, although increases in plasma ALT and AST levels have commonly been used as markers of liver damage [48], both enzymes are also found increased in muscle injury and therefore the elevation may be in part attributed to the affected muscle, rather than to liver or systemic damage [42,57].

At the pathological level, the studies of Cowled and Köksoy have shown that it is also possible to reduce the damage to muscle fibers after IRI-LL. Cowled tested Simvastatin and N-Imino-L-ornithine (L-NIO; a nitric oxide synthase inhibitor,). Though simvastatin alone did not improve the histological assessment of muscle undergoing IRI-LL, L-NIO did significantly reduce it. Moreover, it also induced a significant reduction of neutrophil extravasation and protected against IRI-induced collagen IV degradation, confirming a cytotoxic role of NOS in muscle damage mediated by IRI-LL [16]. However, Köskoy, administering simvastatin (1 mg/kg/day for 6 weeks) to diabetic mice undergoing IRI-LL, demonstrated its therapeutic effect. Pre-treatment with simvastatin led to a 71% reduction in muscle necrosis in diabetic animals, and a 23% improvement in tissue oedema. In addition, skeletal muscle injury, characterized by tissue oedema and the presence of PMNs, was significantly less severe with simvastatin treatment. In this respect, our results were well aligned with those described above [17].

Orban et al. [58] combined N-acetylcysteine (NAC; a well-known antioxidant compound), and preconditioning in patients undergoing knee ligamentoplasty. Although they found no differences in markers of muscle damage, as reported in our work, they noted that the need of narcotic administration for postoperative pain control was significantly reduced with both preconditioning (0.22 ± 0.31 mg/kg vs. 0.47 ± 0.33 mg/kg; *p* < 0.05) and NAC administration (0.22 ± 0.23 mg/kg vs. 0.47 ± 0.33 mg/kg; *p* < 0.05). They also monitored the quadriceps muscle strength of the operated leg with the American Spine Injury Association (ASIA) motor score on the second postoperative day. However, the score obtained in the control group (4.1 ± 0.6) was not improved by preconditioning (4.2 ± 0.8) or NAC treatment (4.3 ± 0.9). Mowafi also demonstrated that melatonin administration (10 mg) reduced postoperative tourniquet-associated pain, as well as a longer time to fentanyl rescue and a reduction in intraoperative fentanyl consumption [14].

Finally, some recent studies have shown that the use of tourniquet in knee surgery worsens postoperative mobility and increases patient-perceived pain. In 2019, Alexandersson et al. reported that three months after undergoing knee surgery, those patients who had not received a tourniquet exhibited 10% greater mobility than those operated under tourniquet, measured by the timed up and go test (TUG) [59]. Ejaz also found similar outcomes in a randomized study with 70 patients, with better function and less pain at the initial rehabilitation stage in those patients in which a tourniquet was not used. Patients without tourniquet showed better performance in both the knee range of motion (ROM) and all subscales of the Knee Injury and Osteoarthritis Outcome Score (KOOS) questionnaire (pain, symptoms, activity of daily living, sport and recreation, and quality of life) [6].

In the present study, to assess how FA could improve motor function in an animal model of IRI-LL, we performed the rotarod test. This test is widely used in other rodent models as a motor ability test [60,61,62,63]. According to the aforementioned clinical studies, we were able to show that, after ischemia, the animals suffered a significant loss of muscle function.

On the following day to ischemia, the time the animals were able to stay on the rod dropped from 200 s to barely (21 s for no treated, and 27 s for FA treated). One week after ischemia, the pre-treated animals had almost reached a “latency to fall time” of 111 s, more 50% of the recorded before ischemia (111 ± 13 s vs. 200 ± 15 s). On the other hand, the control group only achieved a “latency to fall time” of 45% (95 ± 5.8 s vs. 200 ± 15 s). At the end of the experiment (14 days), only the FA group reached pre-ischemia values (211 ± 14 s; *p* > 0.05), while the untreated group just reached 186 ± 6.4 s, a time significantly shorter than the treated animals (*p* < 0.01), and also shorter than the time before ischemia (*p* < 0.05).

## 5. Conclusions

In conclusion, the administration of FA before tourniquet release could be considered as a prophylactic treatment to prevent IRI-LL-induced damage when these devices are used in orthopedic surgery.

## Figures and Tables

**Table 1:** Results of biochemical analysis and levels of electrolytes in serum. Data are presented as the mean and the standard deviation (SD). Alkaline Phosphatase (ALP), Aspartate transaminase (AST), Alanine transaminase (ALT), Creatine kinase (CK), and Lactate dehydrogenase (LDH). Na^+^, K^+^, and Cl^−^ are expressed in milliequivalents per liter (mEq/L), urea and creatinine are expressed in milligrams per deciliter (mg/dL), and ALP, AST, ALT, CK, and LDH are expressed in international units per liter (IU/L).

Parameter	Control Group	Saline 3 h Group	FA 3 h Group	Saline 14 D Group	FA 14 D Group
Na^+^	142 ± 1	143 ± 2	143 ± 2	142 ± 1	142 ± 1
K^+^	3.87 ± 0.29	3.95 ± 0.29	3.97 ± 0.13	4.05 ± 0.3	4.00 ± 0.21
Cl^−^	104 ± 2	100 ± 2	100 ± 3	101 ± 3	100 ± 3
Urea	29.0 ± 3.1	66.3 ± 5.1	59.6 ± 6.1	29 ± 2.8	28.5 ± 2.1
Creatinine	0.36 ± 0.06	0.80 ± 0.13	0.51 ± 0.13	0.41 ± 0.06	0.38 ± 0.04
ALP	123 ± 4	115 ± 5	99 ± 4	85 ± 5	82 ± 13
AST	51.4 ± 2.6	393.2 + 63.6	250.7 ± 20.7	87.0 ± 14.6	59.0 ± 10.5
ALT	41.7 ± 1.6	90.6 ± 4.4	62.0 ± 6.0	31.0 ± 5.2	28.8 ± 4.2
CK	92 ± 15	7792 ± 1187	6123 ± 1000	552 ± 116	298 ± 137
LDH	80 ± 9	1413 ± 227	848 ± 109	1281 ± 339	603 ± 255

**Table 2:** Results of histological analysis of gastrocnemius muscle.

Parameter		Control Group	Saline 24 h Group	FA 24 h Group
Perimeter (mm)	left limb	54.5 ± 2.7	53.8 ± 3.4	54.5 ± 14.9
	right limb	52.8 ± 3.0	61.7 ± 3.4	62.8 ± 2.2
Increase of limb section (ischemic vs. non ischemic) (%)		0.1 ± 7.1	29.9 ± 10.0	17.4 ± 9.2
Weight (g)	left limb	1.82 ± 0.04	1.81 ± 0.14	1.78 ± 0.10
	right limb	1.84 ± 0.09	2.06 ± 0.15	1.86 ± 0.10
PMN (right limb)		8.13 ± 4.93	54.44 ± 19.47	41.80 ± 15.40
Damaged fibers (%)		5.44 ± 2.76	65.53 ± 14.08	48.68 ± 18.86

## Data Availability

The data presented in this study are available in the article.
